# Evolution of *Escherichia coli* rifampicin resistance in an antibiotic-free environment during thermal stress

**DOI:** 10.1186/1471-2148-13-50

**Published:** 2013-02-22

**Authors:** Alejandra Rodríguez-Verdugo, Brandon S Gaut, Olivier Tenaillon

**Affiliations:** 1 Department of Ecology and Evolutionary Biology, UC Irvine, Irvine, CA, USA; 2 INSERM, Université Paris7, Faculté de Médicine Denis Diderot, UMR-S 722, Paris, France; 3 INSERM UMR-S 722, Université Paris7, Faculté de Médicine Denis Diderot, Site Xavier Bichat, 16 rue Henri Huchard, 75018, Paris, France

**Keywords:** Beneficial mutations, Fitness effects, Experimental evolution, Trade-offs, Pleiotropy, Epistasis

## Abstract

**Background:**

Beneficial mutations play an essential role in bacterial adaptation, yet little is known about their fitness effects across genetic backgrounds and environments. One prominent example of bacterial adaptation is antibiotic resistance. Until recently, the paradigm has been that antibiotic resistance is selected by the presence of antibiotics because resistant mutations confer fitness costs in antibiotic free environments. In this study we show that it is not always the case, documenting the selection and fixation of resistant mutations in populations of *Escherichia coli* B that had never been exposed to antibiotics but instead evolved for 2000 generations at high temperature (42.2°C).

**Results:**

We found parallel mutations within the *rpoB* gene encoding the beta subunit of RNA polymerase. These amino acid substitutions conferred different levels of rifampicin resistance. The resistant mutations typically appeared, and were fixed, early in the evolution experiment. We confirmed the high advantage of these mutations at 42.2°C in glucose-limited medium. However, the *rpoB* mutations had different fitness effects across three genetic backgrounds and six environments.

**Conclusions:**

We describe resistance mutations that are not necessarily costly in the absence of antibiotics or compensatory mutations but are highly beneficial at high temperature and low glucose. Their fitness effects depend on the environment and the genetic background, providing glimpses into the prevalence of epistasis and pleiotropy.

## Background

Mutations supply the genetic variation for adaptation, but their success depends on the selective coefficient (*s*), which influences both the probability of fixation of a mutation and its frequency trajectory [1]. Highly advantageous mutations have a higher probability of escaping loss by genetic drift and are also expected to reach high frequency more rapidly than mutations of smaller beneficial effect [2]. The selection coefficient is a function not only of the mutation itself but also of the genetic background; the effect of a mutation may change in amplitude or even in sign (shifting, for example, from beneficial to neutral or even deleterious) across genetic backgrounds due to epistatic effects [3-6]. Epistatic interactions may also limit the emergence and propagation of further beneficial mutations, thereby affecting long-term chances of survival [7]. Finally, *s* may differ across environments (e.g. [8-10]), including the possibility of genotype-by-environment (GXE) interactions. These differential effects may have a profound influence on the pattern of adaptation, because they may prevent a mutation from fixing across heterogeneous environments, which in turn leads to niche (or ecological) specialization [11].

Antibiotic resistance is a particularly important class of beneficial mutation, both because of its potential implications for public health [12] and because resistance is easily studied in the laboratory, particularly in model systems like *Escherichia coli*[13,14]. Genetic resistance to antibiotics can result either from sequential accumulation of multiple beneficial mutations - e.g. resistance to fluoroquinolones [15] – or from a single amino acid substitution - e.g. resistance to rifamycins [16]. This last mutational type is typically highly advantageous in the presence of antibiotics, leading to rapid fixation, often within hundreds of generations [17].

Despite their advantage in the presence of antibiotics, resistance mutations are usually deleterious in the absence of antibiotics, because they often modify vital cellular functions and are highly pleiotropic. For example, amino acid substitutions in the β subunit of the RNA polymerase (RNAP) that produce resistance to rifampicin [18] diminish the transcription efficiency of RNAP and often entail a fitness cost in the absence of rifampicin [19,20]. However, antibiotic resistance may not always be associated with fitness costs; resistance mutations sometimes appear to be neutral or even beneficial in the absence of antibiotics [10,21,22], but for these cases the possibility of secondary advantageous mutations have not been precluded. Costly resistance mutations may also lead to the rapid selection of compensatory mutations that diminish or cancel the cost of resistance [17,20,23,24]. Nonetheless, the paradigm remains that antibiotic resistance is typically selected by the presence of the antibiotic and costly in its absence.

Here we characterize a series of mutations that confer antibiotic resistance but appeared as beneficial mutations in the absence of antibiotics. These mutations arose in the context of an experiment to adapt 114 lines of *E. coli* to thermal stress for 2000 generations [25]. At the end of the experiment, we identified rifampicin resistant clones. Surprised to find their emergence and prevalence in the absence of antibiotics, we have explored the evolutionary context of their appearance, along with the fitness effect of single mutants under different environmental conditions and genetic backgrounds. To do so, we first monitor the trajectory of rifampicin-resistant clones within the evolution experiment, showing that the frequency trajectory varies with the time of first appearance and other factors. We then demonstrate that resistance is conferred by three previously characterized variants and confirm that these mutations confer a high level of fitness advantage under the conditions of the evolution experiment. Finally, we show that these same mutations can be highly deleterious with different genetic backgrounds and environmental conditions, thereby providing glimpses into the prevalence of epistasis and pleiotropy for even well-characterized mutations.

## Methods

### Bacterial strains and experimental design

We examined 114 replicated experimental lines from a previous experiment [25]. The high temperature adapted lines were founded from a common ancestral strain of *E. coli* B (genotype *REL1206*), which was descended from the strain *REL606* after 2000 generations at 37°C in Davis minimal medium supplemented with 25 μg/ml glucose (DM25). The ancestor was evolved in 114 replicate lines at 42.2°C for 2000 generations. Each line was founded from a single colony from an asexual clone (*REL1206*) that was stored at −80°C in a glycerol-based suspension. The lines were propagated by daily transfers of 0.1 ml of each culture into 9.9 ml of DM25, allowing populations sizes to fluctuate daily between 5 x10^6^ cells at the transfer bottleneck to 5 x10^8^ cells, for a total of ~6.64 generations of binary fission per day [26]. Population samples of all 114 lines were taken at 100 generations, 200 generations and at 200-generation intervals thereafter [25].

### Determining the level of rifampicin resistance

To assess the level of antibiotic resistance, we estimated the minimum inhibitory concentration (MICs) of rifampicin (Fisher Scientific, Fair Lawn, NJ) in the ancestral clone and the 114 evolved clones that were characterized genetically [25]. Each isolate was grown in 5 ml of LB broth overnight at 37°C with constant shaking (120 rpm). After diluting the overnight cultures down 10^4^ into MgSO_4_ (10 mM) in a 96 well microplate, we used a multichannel pipette to deliver 2 μL of culture on the surface of the LB agar with rifampicin. The dilution range for rifampicin was 0–800 μg/mL (0, 0.25, 0.5, 1, 2.5, 5, 10, 25, 50, 100, 200, 400, 800). Plates were incubated overnight at 37°C. The MIC was defined as the lowest concentration of antibiotic inhibiting visible growth after overnight incubation. MIC values were confirmed in at least three separate experiments.

We constructed a histogram of the MIC distribution of the *REL1206* ancestor and the 114 evolved lines. A bimodal distribution is usually observed when strains have abnormally elevated MICs, with strains distributed above the upper end of the “susceptible” strains distribution defined as “resistant” strains (Laboratory Standards Institute, CLSI guidelines). Using the data of the first mode (MIC ≤ 10), a log-normal distribution was observed; a MIC of 25 and higher was at least 2.3 standard deviations away from the mean, justifying statistically the resistance status of lines with MIC ≥ 25 and the susceptible status of lines with MIC ≤ 10.

### Fixation parameters and the time of appearance of resistance

We employed mixed population samples from 200-generation intervals to estimate the time of appearance of the rifampicin resistant phenotype. Briefly, cultures were inoculated from frozen stocks into 5 ml LB and incubated overnight at 37°C. 100 μl of the culture was diluted 10^6^-fold and incubated on LB plates at 37°C for 12 hrs. From these plates, we randomly chose 100 colonies to streak on LB plates supplemented with rifampicin at one of three different rifampicin levels - low (12.5 μg/mL), medium (50 μg/mL) and high (100 μg/mL) – corresponding to lower rifampicin concentration than the MIC of the characterized line. We incubated the rifampicin plates at 37°C for 12 hrs and estimated the frequency of resistant individuals in the population by dividing the number of colonies that grew in the LB + rifampicin plates by the number (*n* = 100) of sampled colonies.

Based on the estimated frequency of rifampicin resistance over time, we estimated three parameters of fixation, as inspired by Lang et al. [27]: τ_up_, the time at which mutations reach 1% in the population, starting from the beginning of the experiment; τ_fix_, the time from the beginning of the experiment to the time at which mutations reach 90% in the population; and *s*_up_, the initial rate of increase of mutations (Additional file 1). s_up_ is a proxy for the initial selection coefficient of a rifampicin resistant clone and was measured as the slope of the linear portion between the first two observations of rifampicin resistance in the populations. To estimate these parameters we fitted a linear regression to the natural logarithm of the ratio of resistant vs susceptible over time using the *lm* function in R [28].

### Strain construction and confirmation of recombinants

Single mutations that confer resistance to rifampicin were introduced into the ancestral strain *REL1206* using the pKD46 recombineering plasmid [29]. The pkD46 plasmid carries the lambda Red genes that support homologous recombination. This plasmid also had temperature sensitive replication and an ampicillin resistant marker, so that it can be cured from a strain when grown at 37°C without ampicillin. Briefly, we first introduced the pKD46 plasmid into the ancestral strain, electroporating 1 μl of plasmid (containing between 0.5 and 1 μg of plasmid) into 50 μl of competent cells using an Eppendorf Electroporator 2510 set at 1.8 kV. Following electroporation, we added 1 ml LB and incubated the cells at 30°C for 2 h with shaking. We then plated 100 μl of cells on LB agar plates containing 100 μg/ml ampicillin to select ampicillin-resistant (amp^R^) transformants. The ancestral strain carrying the pKD46 plasmid was then grown overnight at 30°C in 5 ml of LB with 100 μg/ml of ampicillin. The overnight culture was 100 fold-diluted in 100 mL of LB with ampicillin and 1 mM L-arabinose (Sigma) and grown at 30°C to an OD_600_ of 0.6. We made electrocompetent cells by washing the cultures 5 times with ice-cold water.

We designed three oligos of 70 bp with the desired nucleotide change in the center of the oligo (Additional file 2: Table S1) to introduce single point mutations that confer rifampicin resistance. 10 μM of each oligo was electroporated into 50 μl of cells. After electroporation we added 1 ml of LB and incubated cells at 30°C for 16 h with shaking and plated 100 μl in LB agar plates containing rifampicin. We selected single colonies and streaked them onto LB agar plates containing rifampicin. We then incubated the purified colonies on LB broth without antibiotic at 37°C and then tested for ampicillin sensitivity to test for loss of the plasmid. Finally, the correct base replacement was confirmed by Sanger sequencing of ~420 bp of the *rpoB* gene, which was amplified by PCR (Additional file 2: Table S1). The PCR thermal cycling conditions were 95°C for 4 min followed by 30 cycles of 95°C 30 sec and 51°C 30 sec; finally 72°C for 5 min.

### Measurement of relative fitness effects of *rpoB* mutations

The fitness of the single mutant strains relative to the ancestral strain (*A*) was estimated from pairwise competition experiments following standard protocols [26]. Briefly, frozen samples of the mutated and ancestral strains were revived in LB broth and then grown separately for one day at 37°C and a second day at 42.2°C in DM25. The two competitors, a mutant line vs the *A* (*REL1206*) line, were mixed at a 1:1000 volumetric ratio and diluted 100-fold into 10 ml of DM25. We transferred 0.1 ml of each culture mixture daily into 9.9 ml of fresh DM and incubated at 42.2°C over a duration of two days. At the end of the daily growth cycle, we plated 100 μl of the culture on both LB agar plates and LB agar plates supplemented with rifampicin, in order to estimate the density of the total bacterial population (A^rif^ + A) and the resistance density strains (A^rif^), respectively. The frequency of resistant strains (freq A^rif^) was estimated as the density of A^rif^ divided by the density of total population. The relative fitness of the A^rif^ mutants, *w*_*mut*_*,* was determined from the slope of the regression *= ln [freq A*^*rif*^*/(1-freq A*^*rif*^*)]* plotted against the time course in generations [3,30].

### Measurements of fitness effects across genotypes and environments

To measure the fitness effects of rifampicin resistance mutations in different genetic backgrounds, we introduced the *rpoB* mutations into two additional strains: *E. coli* B *REL606* and *E. coli* K12 *MG1655*. These are among the most widely used laboratory strains and are genetically similar, with more than 99% sequence identity over approximately 92% of their genomes. The mutants derived form these strains were also competed against their original strains (*REL606* and *MG1655*), as described above. To test the differential effects of the mutations across genetic backgrounds, we performed a two-way analysis of variance (ANOVA) with genetic background (3 different *E.coli* strains) and genotype (mutations) as fixed effects.

To determine the differential effects of the mutations across environments, we competed mutants against their ancestors in four environments that differed in the temperature of incubation and/or the composition of the medium: 1) DM25 at 37°C, 2) DM1000 (Davis minimal medium supplemented with 1000 μg/ml glucose) at 37°C, 3) DM1000 at 42.2°C and 4) LB at 42.2°C. To test for differential fitness effects across environments, we performed a two-way ANOVA with environment (5 environments) and genotype (3 mutations) as fixed effects. Finally, we used a mixed-effect model to assess the heterogeneity of fitness effects using genotype (mutations) as a random effect and temperature (37°C and 42.2°C) and glucose (25 μg/ml and 1000 μg/ml) as fixed effects. Statistical analyses were performed using the *lm* and *lmer* functions of R [28] for the two-way ANOVAs and the mixed-effect model, respectively.

## Results

### Parallel mutations in the *rpoB* gene conferred different levels of rifampicin resistance

**Figure 1:**
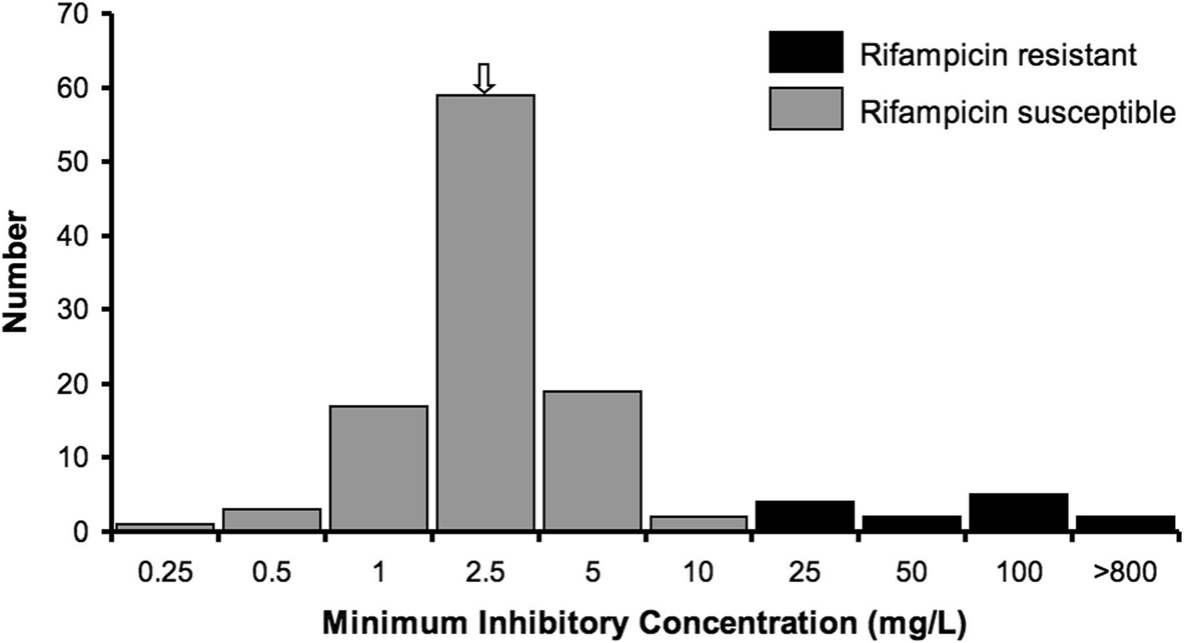
**MIC distribution for the high temperature adapted clones in rifampicin.** The MIC of the ancestral strain is indicated with an arrow.

We screened 114 evolved clones for the presence of rifampicin resistance. These clones represented single isolates from each of the replicate populations at the end of the 2000 generation experiment, and all 114 clones had been sequenced in their entirety [25]. Of these, 13 clones were resistant to rifampicin (Figure 1) at MIC concentrations corresponding to intermediate (25 to 50 μg/ml), high (100 μg/ml) and very high (more than 800 μg/ml) level of rifampicin resistance (Table 1).

**Table 1:** **Non-synonymous mutations in the *rpoB *gene conferring rifampicin resistance**

**High temperature adapted clones**	**Nucleotide change**	**Amino acid change**	**Mutation change**	**Codon change**	**Phenotype (MIC in Rifampicin)**	**Level of rifampicin resistance**
56	ATC → TTC	Ile (I) → Phe (F)	A1714T	I572F	800 μg/mL	VERY HIGH
61	ATC → TTC	Ile (I) → Phe (F)	A1714T	I572F	800 μg/mL	
27	ATC → CTC	Ile (I) → Leu (L)	A1714C	I572L	100 μg/mL	HIGH
35	ATC → CTC	Ile (I) → Leu (L)	A1714C	I572L	100 μg/mL	
92	ATC → CTC	Ile (I) → Leu (L)	A1714C	I572L	100 μg/mL	
97	ATC → CTC	Ile (I) → Leu (L)	A1714C	I572L	100 μg/mL	
142	ATC → CTC	Ile (I) → Leu (L)	A1714C	I572L	100 μg/mL	
4	ATC → AAC	Ile (I) → Asn (N)	T1715A	I572N	50 μg/mL	INTERMEDIATE
43	ATC → AAC	Ile (I) → Asn (N)	T1715A	I572N	25 μg/mL	
77	ATC → AAC	Ile (I) → Asn (N)	T1715A	I572N	25 μg/mL	
112	ATC → AAC	Ile (I) → Asn (N)	T1715A	I572N	50 μg/mL	
131	ATC → AAC	Ile (I) → Asn (N)	T1715A	I572N	25 μg/mL	
59	CGT → CTT	Arg (R) → Leu (L)	G428T	R143L	25 μg/mL	

Because resistance to rifampicin has been documented previously to be caused by single amino acid substitutions on *rpoB*[18], we investigated the relationship between *rpoB* mutations and resistance. Overall, 46 non-synonymous *rpoB* mutations were observed in the original data set of 114 clones, but only 4 *rpoB* mutations were present in the 13 clones that exhibited rifampicin resistance. Three of these four were in codon 572 of *rpoB* (Table 1), which has been previously been shown to be both within the active site and the location of resistance mutations [16]. Twelve of the 13 lines had a non-synonymous mutation in codon 572, representing substitutions between Isoleucine and either Asparagine (*I572N*), Leucine (*I572L*) or Phenylalanine (*I572F*; Table 1). The level of resistance of these 12 clones was perfectly linked to their genotype. Mutations *I572N*, *I572L* and *I572F* corresponded, respectively, to an intermediate (25 to 50 μg/ml), high (100 μg/ml) and very high (more than 800 μg/ml) level of rifampicin resistance (Table 1). Assuming *I572F* is the sole cause of resistance [18], this single mutation in RNAP increased resistance by more than 320 fold relative to the average MIC of susceptible lines (2.5 μg/ml).

The last resistant clone had an intermediate level of resistance and a mutation in codon 143, which is part of the N-terminus of the β-subunit (Table 1). Mutation *R143L* was found in only one clone, while each of the three separate mutations in codon 572 were all found in at least two clones. Since we were interested in evolutionary aspects of resistance, we focused our attention on the three mutations with a clear signal of selection – i.e., those found to have occurred independently in more than one line - namely mutations *I572N*, *I572L* and *I572F*.

### Rifampicin-resistant clones appeared early

**Figure 2:**
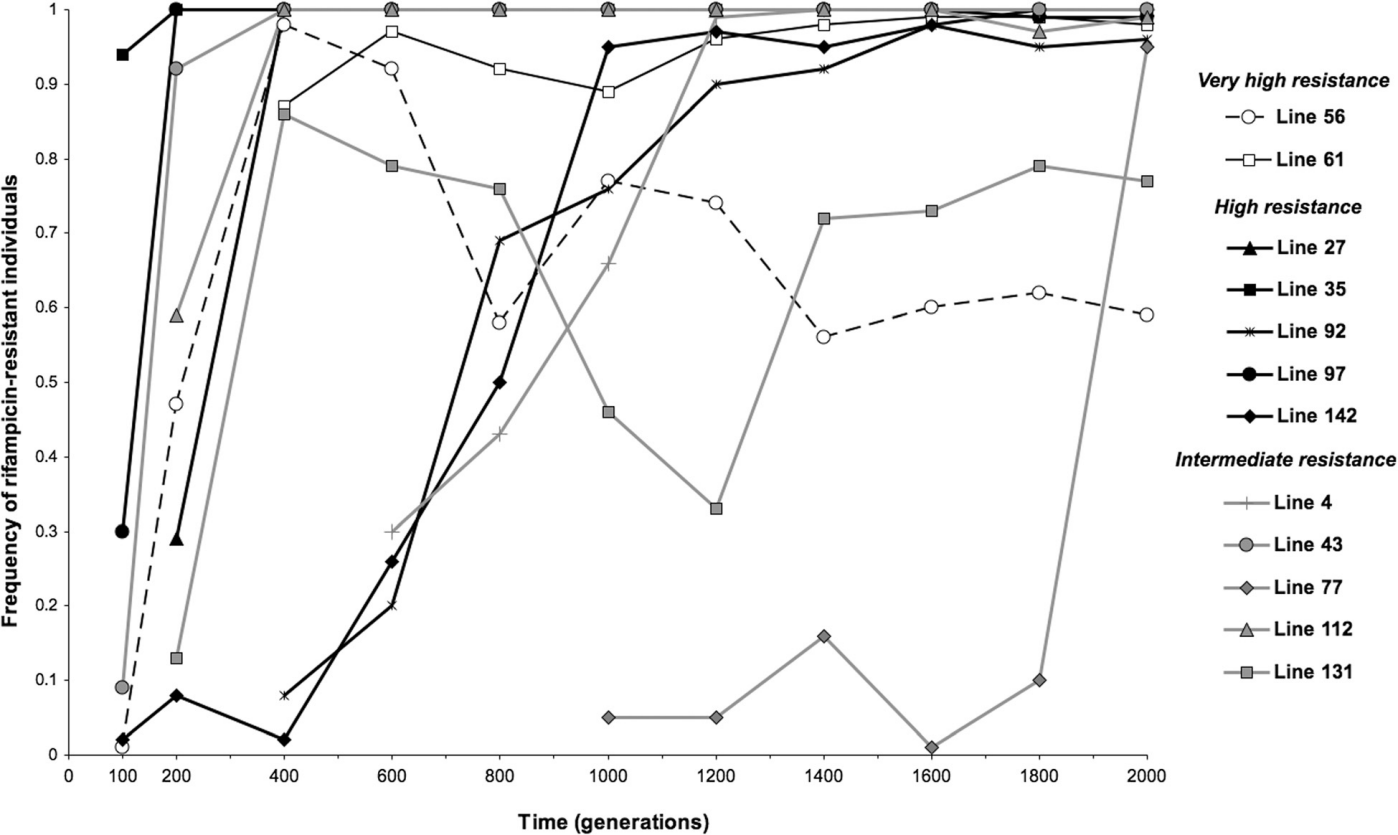
**Temporal dynamics of the rifampicin-resistant individuals in 12 evolved populations during 2000 generations.** The line numbers refer to the high temperature adapted clones in [25].

The 12 rifampicin-resistant clones were chosen randomly for sequencing from their population at the end of the 2000-generation experiment. It was thus unclear if rifampicin resistance was fixed in each of the 12 populations, and it was also unknown when rifampicin resistance appeared during the experiment. To characterize the frequency trajectory of rifampicin resistance, we screened the 12 populations throughout 200 generation intervals (see Methods). As detailed in Figure 2, rifampicin resistance appeared before 500 generations for all 12 lines except line 77, which acquired the resistant phenotype after 800 generations. The resistance phenotype was eventually fixed (f > 0.90) in 10 of the 12 populations; in contrast, resistance appeared early in lines 56 and 131 but did not fix by generation 2000 (Figure 2).

**Figure 3:**
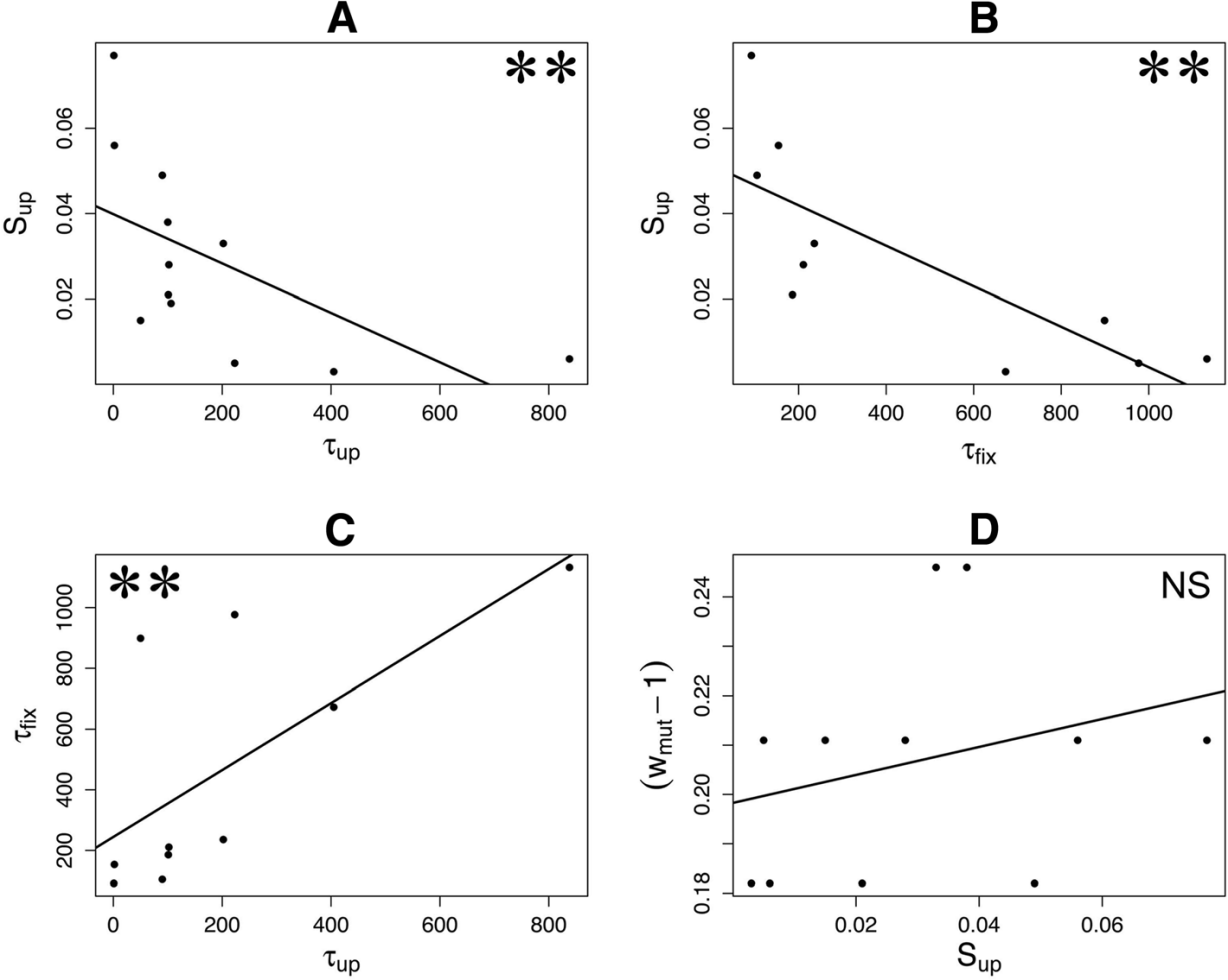
**Relationship between the parameters of fixation. A**) The time at which mutations initially occur (τ_up_) negatively correlates with the initial rate of increase, S_up_ (*P* = 0.0026, Spearman’s rank correlation). **B**) Mutations with lower initial rate of increase (S_up_) take more time to fix (*P* = 0.0016, Spearman’s rank correlation). **C**) Late-occurring mutations (larger τ_up_) take more time to fix (*P* = 0.0092, Spearman’s rank correlation). **D**) No correlation found between the selective advantage measured by direct competition experiments (*w*_*mut*_ – 1) and the initial rate of increase (*P* = 0.3678 Spearman’s rank correlation). For all panels, double asterisks denote significance at *P* < 0.01 and ‘NS’ conveys non-significance (*P* > 0.05).

We estimated parameters of the fixation process from the frequency trajectory of rifampicin resistance, assuming that the resistance has a single origin (Additional files 1 and 3: Table S2). We observed that the time of appearance of resistant mutations (τ_up_) was correlated with the speed and dynamics of fixation (Figure 3). Later-occurring mutations (larger τ_up_) had a smaller initial rate of increase (*s*_up_, Figure 3A), ultimately taking longer to fix (τ_fix_) than early-occurring mutations (Figure 3C). For example, the resistance phenotype in lines 35, 43, 97 and 112 - in which the rifampicin mutants reached a frequency higher than 0.2 at generation 200 (Figure 2) - fixed more rapidly than lines 4, 92 and 142 (Figure 2). Not surprisingly, *s*_up_ was negatively correlated with τ_fix_, so that lines with slower initial rate of increase of the resistance phenotype took longer to fix the phenotype (Figure 3B).

### Mutations in the *rpoB* gene confer both resistance and a selective advantage

To measure the phenotype and selective advantage of *rpoB* non-synonymous mutations in codon 572, we introduced single nucleotide substitutions into the ancestral background (*REL1206*). With these genetic constructions we confirmed that the single amino acid substitutions in codon 572 fully explained the level of rifampicin resistance; that is the *I572N* mutation resulted in intermediate resistance (25 to 50 μg/ml), with high resistance (100 μg/ml) for *I572L* and the highest resistance (800 μg/ml) for *I572F*. Thus, as expected [18], single base mutations in codon 572 of *rpoB* are sufficient to explain the rifampicin resistance phenotype.

**Figure 4:**
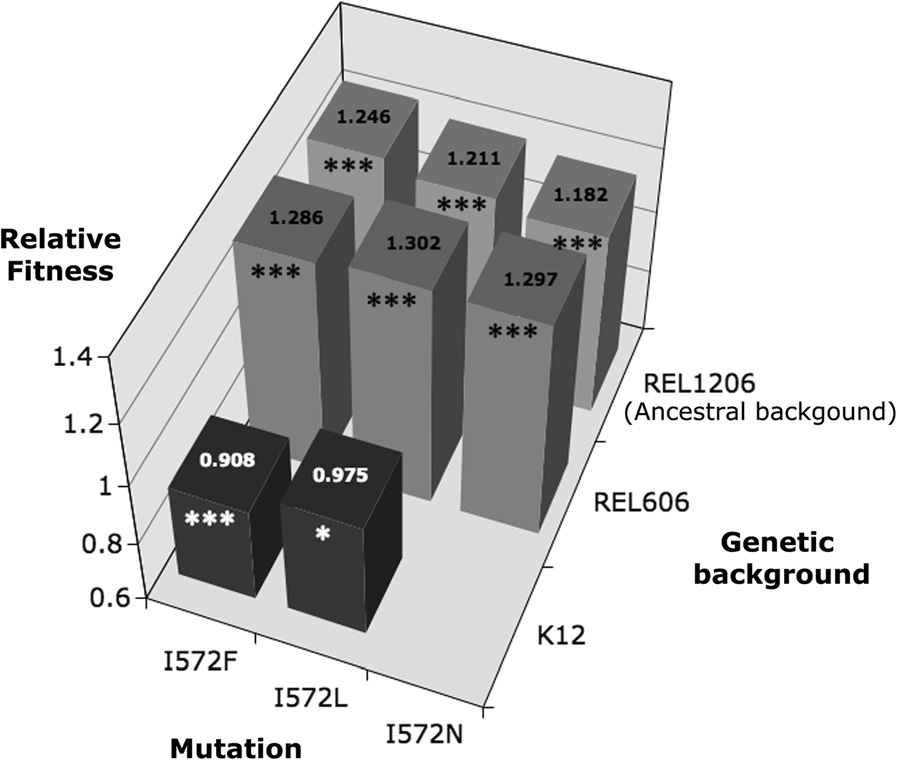
**Relative fitness of the *rpoB *mutants measured at 42.2°C in DM25 in different genetic backgrounds.** The mean relative fitness values, calculated from 6 replicates, are indicated on top of the bars. The gray color corresponds to an advantageous fitness effect of the mutations in relation to the ancestor and the dark color corresponds to a deleterious fitness effect of the mutations in relation to the ancestor. The asterisks represent significant deviation from the null hypothesis that mean fitness equals 1.0, with one and three asterisks denoting significance at *P* < 0.05 and *P* < 0.001, respectively.

We measured the fitness effect of each of the three mutations in competition experiments at 42.2°C in DM25. The three amino acid substitutions conferred (individually) a fitness advantage ranging from 0.182 to 0.246 (Figure 4) relative to the *REL1206* ancestral line. The selective advantage was significantly different among mutations (*P* = 0.0493; ANOVA), with the pairwise difference between being significant between mutations *I572F* and *I572N* (*P =*0.0398; Tukey’s). Thus, the mutations confer resistance to rifampicin as well as a fitness advantage in the absence of rifampicin at 42.2°C in DM25.

### The *rpoB* mutations have differential effects across genotypes and environments

Resistance mutations are usually thought to incur a cost in the absence of antibiotic, but that is not the case for our mutations under the conditions of the original thermal stress experiment. Given the large ~20% selective advantage of these mutations, why is rifampicin resistance not fixed throughout *E. coli sensu lato*? Because previous work has demonstrated a cost to rifampicin resistance in the absence of antibiotic [19,20,31], we suspected differential effects of the codon 572 mutations with respect to genetic background and environmental conditions. We thus assessed the fitness of the mutations in different genetic backgrounds and environments.

To test the effect of genetic background, we inserted the three codon 572 mutations in two additional strains: *E. coli* B *REL606* and *E.coli* K12 *MG1655*. Both are commensal *E.coli* laboratory strains from phylogenetic subgroup A that were isolated a century ago. All three mutations (*I572N*, *I572L* and *I572F*) conferred resistance to rifampicin in the two new backgrounds (*REL606* and *MG1655*; data not shown). However, under thermal stress (42.2°C) and low glucose (DM25) conditions in the absence of rifampicin, the mutations had differential fitness effects depending on genetic background. The mutations were strongly beneficial in *REL606*, with a net fitness benefit similar to that of the *REL1206* background (Figure 4, Additional file 4: Table S3) but deleterious in the K12 background, with a ~2.5% to 10% fitness cost in the single mutants *I572L* and *1572 F* relative to the non-mutated K12 *MG1655* competitor. In fact, despite several attempts, we were unable to introduce the *I572N* mutation into the *MG1655* background, suggesting that the *I572N* mutation may have a fitness of zero (lethality) in the *MG1655* background. Consistent with these fitness observations, an analysis of variance detected a significant effect of genetic background (Table 2). Although we did not detect a significant background-by-mutation interaction, the low *P-*value (0.053) suggests that the three mutations have differential effects across genetic backgrounds.

**Table 2:** Two-way analysis of variance for relative fitness of mutants in three different genetic backgrounds

**Analysis of variance**					
Source	df	SS	MS	F values	P
Background	2	1.23909	0.61955	207.9335	<0.0001***
Genotype (Mutation)	1	0.00737	0.00737	2.4734	0.12367
Background x Genotype	2	0.01881	0.00941	3.1568	0.05334
Residuals	40	0.11918	0.00298		

We treated the genetic background and the genotype (mutation) as fixed effects.

**Figure 5:**
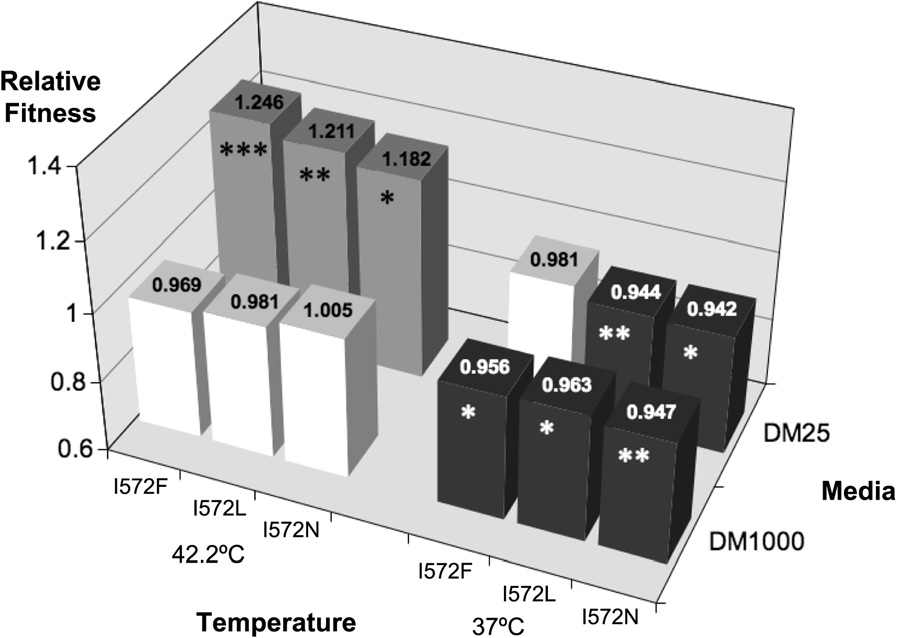
**Relative fitness of the *rpoB *mutants measured in different conditions.** The mean relative fitness values are indicated in the top of the bars. The gray color corresponds to an advantageous fitness effect of the mutations in relation to the ancestor, the dark color corresponds to a deleterious fitness effect of the mutations in relation to the ancestor, and the white color corresponds to a neutral fitness effect of the mutations in relation to the ancestor. The asterisks represent a significant difference from a mean fitness of 1.0, based on one-tailed t-distribution with n-1 degrees of freedom. One asterisk represents significance at *P* < 0.05; two denote significance at *P* < 0.01; three convey significance at *P* < 0.001.

We also assessed relative fitness for mutations in *REL1206* background for five different environments, including two glucose treatments (DM25 and DM1000), two temperatures (37.0°C and 42.2°C), and a different medium (LB). These experiments revealed that fitness effects varied across environments (Figure 5, Table 3). The three *rpoB* mutations were costly at 37°C in DM25, at 37°C in DM1000 and at 42.2°C in LB medium (Additional file 4: Table S3), but the relative fitness did not differ from neutrality (i.e., a relative fitness that differs from 1.0) at 42.2°C in DM1000. There was also a significant non-additive interaction between temperature and the concentration of glucose in DM media (Table 3); that is, the deleterious effect on fitness of both conditions (DM1000, 37°C) was not the sum of the deleterious effect on fitness of each condition separately.

**Table 3:** Two-way analysis of variance for relative fitness of mutants in five different environments with the environment and the genotype (mutants) treated as fixed effects, and a mixed effect model with the genotype treated as random and the temperature and glucose treated as fixed effects

**Analysis of variance**					
Source	df	SS	MS	F values	P
Environment	4	1.18349	0.295871	49.0517	<0.0001***
Genotype	2	0.00386	0.001930	0.3200	0.7268
Environment x Genotype	8	0.05311	0.006639	1.1006	0.3679
Residuals	117	0.70572	0.006032		
**Mixed effects model**					
Source	df	F values	P		
Temperature	1	49.171	<0.0001***		
Glucose	1	49.333	<0.0001***		
Temperature x Glucose	1	52.560	<0.0001***		

## Discussion

The basis for our study is the observation that rifampicin resistance arose in the absence of an antibiotic during an evolutionary experiment. After 2000 generations of thermal stress, 13 of 114 *E. coli* clones exhibited resistance to rifampicin. Twelve of these 13 clones included a mutation in codon 572 of the *rpoB* gene, with three different mutations observed in that codon (Table 1). These three mutations have been noted previously to confer rifampicin resistance [32], a finding we have reconfirmed. Moreover, each of these three mutations occurred independently in more than one population, providing strong evidence by the criterion of evolutionary convergence [33] that the mutations are beneficial under the experimental conditions. Concerning the 13th and final clone, a mutation in codon 143 has been previously described to confer low resistance to rifampicin (*R143W*, [34]), but the mechanistic causes of resistance for this clone remain unclear. However, our analysis of the RNAP 3-D structure suggests that codon 143 folds into the vicinity of the active (i.e., binding) site of the RNAP (Additional file 5). It is possible, then, that mutations in this codon alter rifampicin binding, thus leading to resistance.

We used both direct and indirect evidence to confirm that all three mutations in codon 572 result in a fitness advantage within a thermal stress/low glucose environment. For direct evidence, we introduced single mutations into the ancestral *REL1206* background and assessed the relative fitness of mutants to unmutated *REL1206*. The measured fitness effect varied statistically among the three mutations, with relative fitnesses ranging from 1.18 to 1.25. Perhaps the most notable feature of these measurements is the magnitude of the effect. In the experimental evolution literature, it is rare to find single mutations with relative fitness benefits above ~15% [35,36]. Thus, with the exception of mutations that compensate the cost of antibiotic resistance [35], the measured fitness benefits of the single *I572N*, *I572L* and *I572F* mutations are uncommon [6,37]. We note, however, that these high fitness values still explain only a fraction of the total realized relative fitness benefit of the twelve evolved clones, which have accumulated an average of 8 mutations compared to *REL1206* and an average relative fitness increase of ~40% (mean relative fitness 1.396; stdev 0.122) [25].

Indirect evidence for the benefit of these mutations comes from the assessment of the frequency trajectory of rifampicin resistance over the course of the full 2000-generation experiment. Generally, rifampicin resistance evolved early – within 500 generations - and swept to fixation within a few hundred generations (Figure 2). This steep increase in frequency is consistent with a high selection coefficient for the haplotypes that carry the resistance marker. We have measured the selection coefficient for these haplotypes by estimating *s*_up_, which ranges between 0.015 and 0.077 (Figure 3A, Additional file 3: Table S2). While these are high selection coefficients, they are not directly comparable to our relative fitness estimates, for several reasons (see below). What *s*_up_ does, however, is confirm that the capability for antibiotic resistance may be highly beneficial even in the absence of antibiotic.

### Fixation dynamics of the resistance mutations

The frequency trajectories also provide crucial insights into the fixation dynamics of beneficial mutations. One interesting observation is that the relative fitnesses of single rif^R^ mutations do not correlate with the estimated selective coefficient (*s*_up_) of the populations that harbor these mutations (Figure 3D). This may reflect a lack statistical power to detect a correlation - since there are only three relative fitness measures – or may reflect the possibility that the resistant individuals observed at intermediate time points harbor different resistant mutations than the one observed at the end of the experiment. Nonetheless, we believe the lack of a relationship is meaningful. For example, the *I572F* rifampicin-resistant mutation found in lines 56 and 61 has the highest relative fitness as a single mutation (Figure 5), but rifampicin resistance was not fixed rapidly in these two lines. Instead, we find that early-occurring rif^R^ mutations take less time to reach fixation than late-occurring mutations (Figure 3C); this pattern suggests either that epistasis, clonal interference or frequency dependent fitness interactions influences *s*_up_[27].

For the former (epistatic interactions), diminishing-returns epistasis is expected theoretically [4] and has been observed empirically as more and more mutations accumulate over the time-course of an experiment [6,37]. Under diminishing-returns, a relatively late occurring *rpoB* mutation may have a smaller fitness effect, conditional on the occurrence of previous beneficial mutations. For clonal interference, competition between beneficial haplotypes will slow the process of fixation [38,39]. Finally, complex dynamics such as those observed in lines 56 and 131 might be due to frequency-dependent selection. In any case, such competition may be more common in the later stages of an experiment when multiple mutations have accrued [38]. In contrast, early rif^R^ mutations likely occurred in a *REL1206* background that was fairly devoid of other new mutations, thus minimizing possibilities for either clonal interference or epistatic interactions with other new mutations.

The possibility that frequency trajectories have been shaped in part by epistasis (whether as diminishing-returns or one of several other possible forms [40]) is not surprising given the study of Tenaillon et al. [25]. This study detected statistical associations among mutations that were consistent with extensive and varied epistatic effects. These associations shaped the adaptive response to thermal stress into one of two distinct genetic solutions typified by mutations either in *rpoB* or in the termination factor *rho*, but rarely in both genes. To investigate the potential relationship of *s*_up_ to these statistical associations, we examined genetic data from Tenaillon et al. [25]. Clones from lines 43, 61 and 131, all of which had high τ_fix_ values (> 400 generations; Figure 2) carried mutations in both *rho* and *rpoB*, a combination statistically highly disfavored among the full dataset of 114 clones. This observation suggests that the long fixation time in these lines could be due in part to negative epistatic interactions between *rho* and *rpoB* mutations that reduces beneficial effects of both mutations. The strength and mechanism of these interactions need to be characterized more fully, however.

Previous studies have identified potential epistatic interactions with mutations in codon 572 [41], and epistasis must contribute to varying fitness effects among our genetic backgrounds (Figure 4). In the high temperature and low glucose condition, our three codon 572 mutations conferred a slightly (but not significantly) higher relative fitnesses in the *REL606* background than in the ancestral *REL1206* background. The similar effects in these two backgrounds may not be surprising, however, given that *REL1206* and *REL606* differ by only a handful of mutations: *REL1206* differs from *REL606* in 3 SNPs, an IS element and a large deletion [25,42]. In contrast, the rif^R^ mutations are detrimental in the K12 *MG1655* background (Table 2), even though K12 and B are genetically similar (> 99% sequence identity over ~92% of their genomes [43]).

### The specificity of adaptation

The effects of the rifampicin resistance mutations also vary as a function of environment. In our study, the only environment in which the mutations are demonstrably beneficial is that of the original evolution experiment (high temperature and low glucose). In contrast, the effects of rif^R^ mutations are indistinguishable from neutrality in a high temperature and rich glucose environment and demonstrably detrimental at 37°C in poor and rich glucose environment [19,20,31] (Figure 5, Additional file 4: Table S3).

With the exception of lethal selection or niche creation [11] experiments, most other studies have demonstrated that the fitness advantage conferred by a mutation is maintained across environments and conditions [9,10]. In other words, they have found that beneficial mutations are generally not severely compromised in other environments [10]. The logical extension of these observations is that a single beneficial mutation is unlikely to result in niche specialization, because it will not lead to drastic fitness differences across environments.

In stark contrast to these studies, we do observe the potential for the evolution of ecological specialization in a single mutational step, because all three mutations in codon 572 of *rpo*B confer a selective advantage in the conditions of the original evolution experiment but significant disadvantages in other environments (Table 3) and genetic backgrounds (Table 2). In this context, it is important to repeat that this potential for niche specialization is not a function of antibiotic resistance, for which niche specialization is well known, but rather due to fitness effects across antibiotic-free environments.

The question remains as to whether our single *rpo*B mutations are rare or instead cast doubt on previous conclusions that niche specialization is “… unlikely to occur through the substitution of a single mutation” [10]. The degree of ecological specialization for our single mutations could be due in part to the drastic selection pressure (high temperature) in the original experiment or to *rpoB* itself. Because mutations within *rpoB* can be highly pleiotropic, they can affect a series of downstream traits like gene expression [44,45] that may be fine-tuned for specific selective regimes. We note that highly pleiotropic (but non-*rpo*B) mutations have been observed in early stages of adaptation to ethanol stress [46] and glycerol minimal media [47], suggesting that early mutations in adaptation are commonly involved in transcriptional regulation with large fitness and pleiotropic effects [48,49]. As such, our *rpoB* mutations may not be uncommon, either in their effects or in their potential for ecological specialization. Thus, in our opinion, the frequency and occurrence of niche-specialization by single beneficial mutations is still an open question worthy of further study.

### Mechanism

Several experimental evolution studies have shown the fixation of mutations in RNAP during stress adaptation [44,45]. This suggests that modifications in the RNAP could be a general mechanism for adaptation to new environments. Yet, the mechanistic basis for the beneficial effect of *rpoB* mutations at high temperature remains unclear. Since temperature affects the stability and activity of proteins [50-52], *rpoB* mutations may modify the stability and/or activity of RNAP at high temperatures. For example, previous studies have shown that mutation *I572F* increase transcription termination [53], and mutation *I572L* reduces transcription efficiency at 37°C [19]. Another (but not mutually-exclusive) hypothesis is that *rpoB* mutations cause changes in gene expression through the redistribution of RNAP in manner that favors adaptation to new environments [44]. The unique challenge here is explaining how these mechanistic effects can be advantageous in *REL1206* but (for example) disadvantageous in K12 (Figure 4). Fortunately, questions of mechanism are amenable to future experimental investigation.

## Conclusions

Numerous studies have investigated rifampicin resistance in bacterial populations. The general tenor of these studies is that rifampicin resistance is deleterious in the absence of an antibiotic, and thus compensatory mutations are required for resistance to persist (e.g. [20]). Our study differs from most previous in demonstrating the origin of resistance in the absence of antibiotics and also in demonstrating that the resistance mutations can be highly beneficial in the absence of antibiotic, depending on both the background of the mutation and the environment.

## Competing interests

The authors declare that they have no competing interests.

## Authors’ contributions

AR-V helped designing the study, performed the experiments and analyses. BG participated in the design of the study and in the analysis of data. OT conceived and designed the study. All authors participated in writing the paper and they all approved the final manuscript.

## Supplementary Material

Additional file 1 Parameters of fixation. Click here for file

Additional file 2 Table S1 Oligonucleotides and primers used in this study. Click here for file

Additional file 3 Table S2 Parameters of fixation estimated from the frequency trajectories. Click here for file

Additional file 4 Table S3 Relative fitness measured in different conditions and different genetic backgrounds. Click here for file

Additional file 5 **Three-dimensional structure of RNAP generated using Jmol from the Protein Data Bank (**http://www.rcsb.org**).** Click here for file
