# Supplementary figures and images for: Evolution of Escherichia coli rifampicin resistance in an antibiotic-free environment during thermal stress
Source: BMC Evol Biol. 2013 Feb 22;13:50. doi: 10.1186/1471-2148-13-50 (PMC3598500; doi:10.1186/1471-2148-13-50)

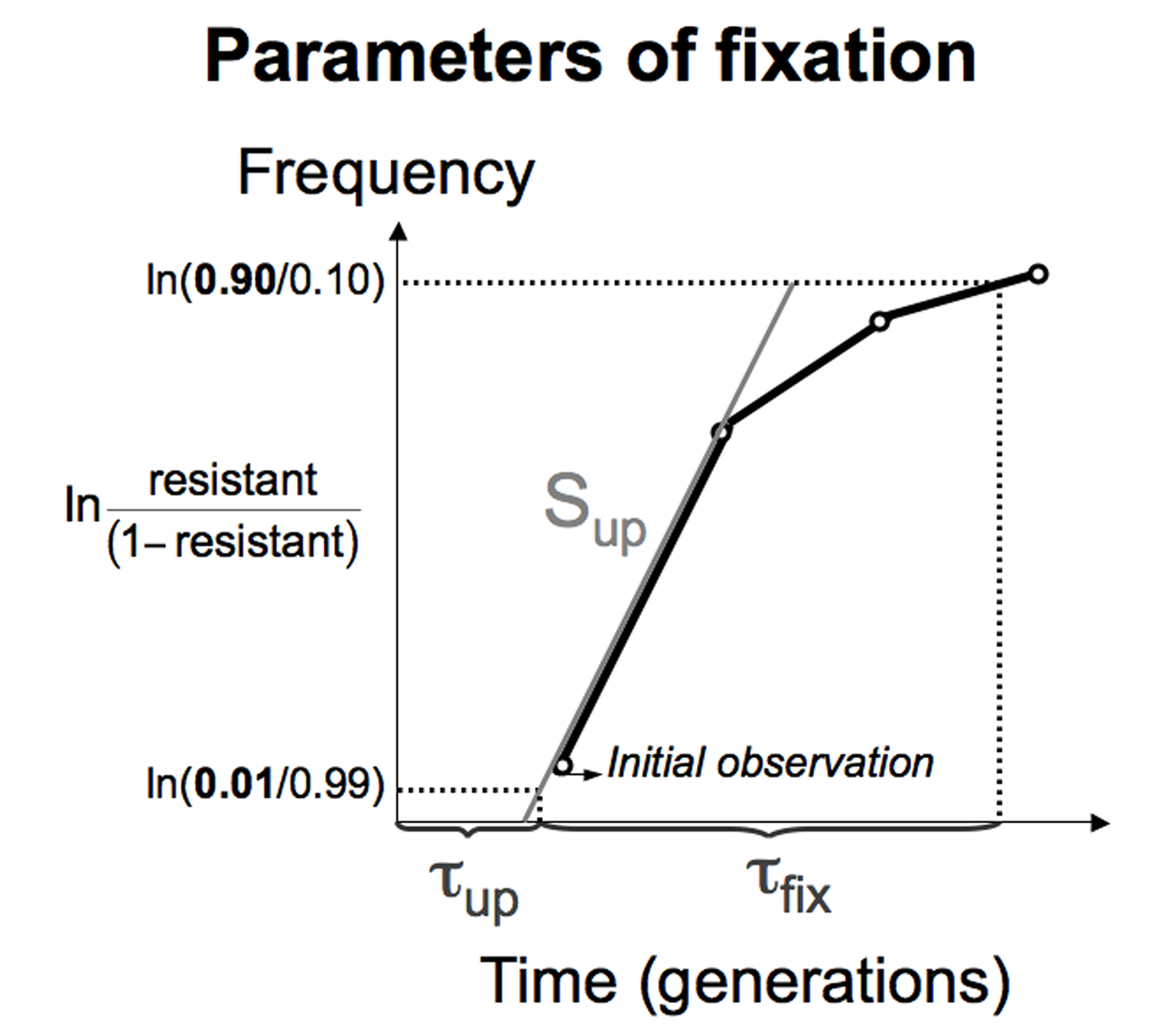

Supplement: Additional file 1 — Parameters of fixation. [file 1471-2148-13-50-S1.tiff]

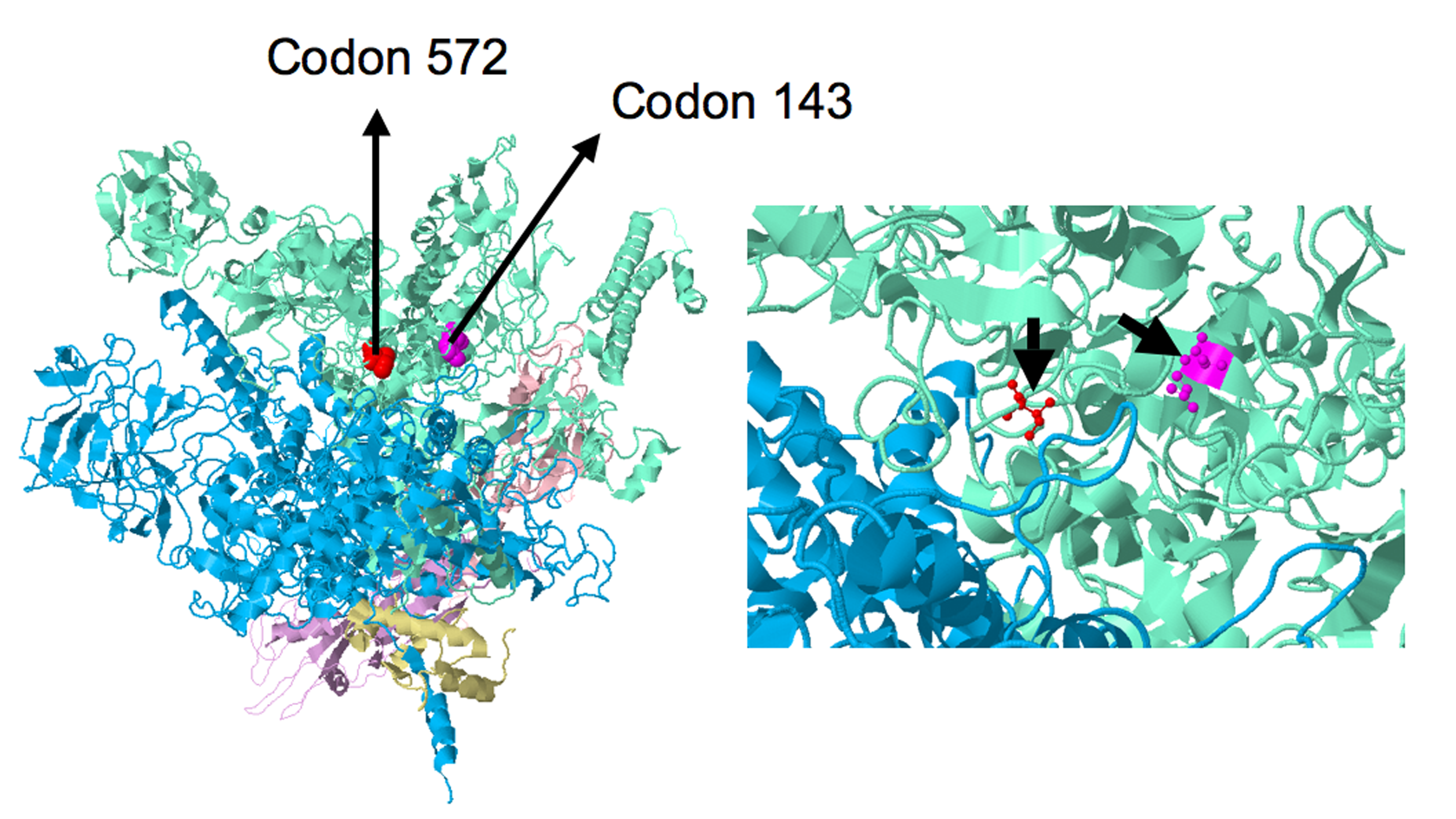

Supplement: Additional file 5 — Three-dimensional structure of RNAP generated using Jmol from the Protein Data Bank (http://www.rcsb.org). [file 1471-2148-13-50-S5.tiff]
