# Supplementary material for: Evolution of Escherichia coli rifampicin resistance in an antibiotic-free environment during thermal stress
Source: BMC Evol Biol. 2013 Feb 22;13:50. doi: 10.1186/1471-2148-13-50 (PMC3598500; doi:10.1186/1471-2148-13-50)
Supplement: Additional file 2 Table S1 — Oligonucleotides and primers used in this study. [file 1471-2148-13-50-S2.pdf]

Table S1. Oligonucleotides and primers used in this study.

Oligos used for recombineering to create *rpoB* mutants

I572F	5'- ATC GAA ACC CCT GAA GGT CCG AAC ATC GGT CTG TTC AAC TCT CTG TCC GTG TAC GCA CAG ACT AAC GAA TA -3'
I572L	5'- ATC GAA ACC CCT GAA GGT CCG AAC ATC GGT CTG CTC AAC TCT CTG TCC GTG TAC GCA CAG ACT AAC GAA TA -3'
I572N	5'- ATC GAA ACC CCT GAA GGT CCG AAC ATC GGT CTG AAC AAC TCT CTG TCC GTG TAC GCA CAG ACT AAC GAA TA -3'

Primer pair used for PCR amplification of *rpoB* region

I572 Forward	5'- ACA ACC CGC TGT CTG AGA TT -3'
I572 Reverse	5'- TGG GTG GAT ACG TCC ATG TAG -3'
