# Supplementary material for: Evolution of Escherichia coli rifampicin resistance in an antibiotic-free environment during thermal stress
Source: BMC Evol Biol. 2013 Feb 22;13:50. doi: 10.1186/1471-2148-13-50 (PMC3598500; doi:10.1186/1471-2148-13-50)
Supplement: Additional file 3 Table S2 — Parameters of fixation estimated from the frequency trajectories. [file 1471-2148-13-50-S3.pdf]

Table S2. Parameters of fixation estimated from the frequency trajectories.

High temperature adapted lines	Codon change	Phenotype (MIC in Rifampicin)	S_{up}	τ_{up} (generations)	τ_{fix} (generations)
35	I572L	100 μg/mL	0.077	1	92
97	I572L	100 μg/mL	0.056	2	154
43	I572N	25 μg/mL	0.049	90	105
56	I572F	800 μg/mL	0.038	100	Not fixed
61	I572F	800 μg/mL	0.033	202	236
27	I572L	100 μg/mL	0.028	102	211
112	I572N	50 μg/mL	0.021	101	186
131	I572N	25 μg/mL	0.019	106	Not fixed
142	I572L	100 μg/mL	0.015	50	899
77	I572N	25 μg/mL	0.006	838	1133
92	I572L	100 μg/mL	0.005	223	977
4	I572N	50 μg/mL	0.003	405	673
