# Supplementary material for: Evolution of Escherichia coli rifampicin resistance in an antibiotic-free environment during thermal stress
Source: BMC Evol Biol. 2013 Feb 22;13:50. doi: 10.1186/1471-2148-13-50 (PMC3598500; doi:10.1186/1471-2148-13-50)
Supplement: Additional file 4 Table S3 — Relative fitness measured in different conditions and different genetic backgrounds. [file 1471-2148-13-50-S4.pdf]

Table S3. Relative fitness measured in different conditions and different genetic backgrounds.

Genetic background	Fitness assay at	Mutant	Codon Change	Relative fitness (±95%CI)[1]	p-value[2]	n	Phenotypic effect
REL 1206 (Ancestral background)	42.2°C in DM25	A^{Rif} 1	I572F	1.246 ± 0.012	**<0.0001∗∗∗**	6	**24.6% Advantage**
		A^{Rif} 2	I572L	1.211 ± 0.018	**<0.0001∗∗∗**	6	**21.1% Advantage**
		A^{Rif} 3	I572N	1.182 ± 0.020	**0.0001∗∗∗**	6	**18.2% Advantage**
	37°C in DM25	A^{Rif}1	I572F	0.981 ± 0.013	0.0893	12	Neutral
		A^{Rif}2	I572L	0.944 ± 0.017	**0.011∗**	6	**5.6% Cost**
		A^{Rif}3	I572N	0.942 ± 0.016	**0.0064∗∗**	6	**5.8% Cost**
	42.2°C in DM1000	A^{Rif}1	I572F	0.969 ± 0.055	0.2944	12	Neutral
		A^{Rif}2	I572L	0.981 ± 0.015	0.1155	12	Neutral
		A^{Rif}3	I572N	1.005 ± 0.017	0.6065	12	Neutral
	37°C in DM1000	A^{Rif}1	I572F	0.956 ± 0.017	**0.0135∗**	12	**4.4% Cost**
		A^{Rif}2	I572L	0.963 ± 0.015	**0.0147∗**	12	**3.7% Cost**
		A^{Rif}3	I572N	0.947 ± 0.017	**0.0051∗∗**	12	**5.3% Cost**
	42.2°C in LB	A^{Rif}1	I572F	0.868 ± 0.014	**0.0001∗∗∗**	6	**13% Cost**
		A^{Rif}2	I572L	0.836 ± 0.033	**0.002∗∗**	6	**13% Cost**
		A^{Rif}3	I572N	0.928 ± 0.030	**0.03∗**	6	**9.8% Cost**
REL 606	42.2°C in DM25	R^{Rif}1	I572F	1.286 ± 0.030	**<0.0001∗∗∗**	6	**28.6% Advantage**
		R^{Rif}2	I572L	1.302 ± 0.036	**0.0002∗∗∗**	6	**30.2% Advantage**
		R^{Rif}3	I572N	1.297 ± 0.038	**0.0003∗∗∗**	6	**29.7% Advantage**
K12 (MG1655)	42.2°C in DM25	K^{Rif}1	I572F	0.908 ± 0.018	**0.0004∗∗∗**	6	**9.2% Cost**
		K^{Rif}2	I572L	0.975 ± 0.010	**0.0218∗**	6	**2.5% Cost**

[1]Mean relative fitness and 95% confidence interval based on n replicates.

[2]Significance values based on one-tailed t-distribution with n-1 degrees of freedom; the null hypothesis is that the mean fitness equals 1.
